# Single Nucleotide Polymorphisms Interactions of the Surfactant Protein Genes Associated With Respiratory Distress Syndrome Susceptibility in Preterm Infants

**DOI:** 10.3389/fped.2021.682160

**Published:** 2021-10-04

**Authors:** Shaili Amatya, Meixia Ye, Lili Yang, Chintan K. Gandhi, Rongling Wu, Beth Nagourney, Joanna Floros

**Affiliations:** ^1^Department of Pediatrics, Center for Host Defense, Inflammation, and Lung Disease (CHILD) Research, Pennsylvania State University College of Medicine, Hershey, PA, United States; ^2^Center for Computational Biology, College of Biological Sciences and Technology, Beijing Forestry University, Beijing, China; ^3^School of First Clinical Medicine, Nanjing University of Chinese Medicine, Nanjing, China; ^4^Public Health Science, Pennsylvania State University College of Medicine, Hershey, PA, United States; ^5^Albert Einstein College of Medicine, New York, NY, United States; ^6^Obstetrics and Gynecology, Pennsylvania State University College of Medicine, Hershey, PA, United States

**Keywords:** epistasis, neonatal, genetic variants, pulmonary, allele

## Abstract

**Background:** Neonatal respiratory distress syndrome (RDS), due to surfactant deficiency in preterm infants, is the most common cause of respiratory morbidity. The surfactant proteins (*SFTP*) genetic variants have been well-studied in association with RDS; however, the impact of SNP-SNP (single nucleotide polymorphism) interactions on RDS has not been addressed. Therefore, this study utilizes a newer statistical model to determine the association of *SFTP* single SNP model and SNP-SNP interactions in a two and a three SNP interaction model with RDS susceptibility.

**Methods:** This study used available genotype and clinical data in the Floros biobank at Penn State University. The patients consisted of 848 preterm infants, born <36 weeks of gestation, with 477 infants with RDS and 458 infants without RDS. Seventeen well-studied *SFTPA1, SFTPA2, SFTPB, SFTPC*, and *SFTPD* SNPs were investigated. Wang's statistical model was employed to test and identify significant associations in a case-control study.

**Results:** Only the rs17886395 (C allele) of the *SFTPA2* was associated with protection for RDS in a single-SNP model (Odd's Ratio 0.16, 95% CI 0.06–0.43, adjusted *p* = 0.03). The highest number of interactions (*n* = 27) in the three SNP interactions were among *SFTPA1* and *SFTPA2*. The three SNP models showed intergenic and intragenic interactions among all *SFTP* SNPs except *SFTPC*.

**Conclusion:** The single SNP model and SNP interactions using the two and three SNP interactions models identified *SFTP*-SNP associations with RDS. However, the large number of significant associations containing *SFTPA1* and/or *SFTPA2* SNPs point to the importance of *SFTPA1* and *SFTPA2* in RDS susceptibility.

## Introduction

Neonatal respiratory distress syndrome (RDS) is the most common cause of respiratory failure in premature infants due to surfactant deficiency (1). However, the infant mortality rate due to RDS was 11.4 per 100,000 live births and accounted for 2% of all infant deaths in 2017 in the United States (2) despite the judicious use of postnatal surfactant along with antenatal steroids (3).

Major risk factors, such as prematurity and low birth weight (BW) along with sex and race (4–7) have been implicated in RDS. Genetic factors have also been associated with RDS by various twins' studies (8, 9). Thus, the susceptibility to RDS is considered multifactorial and/or polygenic (10), with ample evidence in the literature that gene–host-environment interactions may play a large role in the morbidity and mortality associated with this syndrome. The understanding of gene interactions in RDS may help identify novel therapeutic targets for susceptible infants.

Furthermore, it has been noted that infants dying with RDS have low levels of surfactant proteins (SP) (11, 12). SP-A and SP-D are hydrophilic proteins and play an important role in innate immunity and the regulation of inflammatory processes and host defense (13–17). SP-B and SP-C are hydrophobic proteins that enhance the adsorption and spreading of surfactant phospholipid (18). In addition, SP-B is essential for lung function by reducing surface tension and preventing alveolar collapse (19–21). SP B and SP-C are present in the exogenous surfactant used to treat RDS. However, SP-A and SP-D (SP-D co-isolates with the surfactant complex) are not included in the formulation, even though a major complication in prematurely born infants with RDS is infection. In addition to its host defense function, SP-A, along with SP-B, is important for the formation of tubular myelin (an extracellular surfactant structure) (22–24). Moreover, SP-A is involved in surfactant-related functions (17, 25) and lung airway function (26).

Multiple genetic variants and single nucleotide polymorphisms (SNP) of the surfactant protein gene (*SFTP)* have been shown to associate with RDS (10, 27–40). Human SP-A, consisting of SP-A1 and SP-A2 proteins, is encoded by two functional genes *SFTPA1* and *SFTPA2*, respectively (41). The *SFTPA1* and *SFTPA2* genes share a high degree of sequence similarity but differ at various splice variants at the 5′ untranslated region (UTR) and exhibit sequence variability within coding and non-coding regions (17). Prior studies have also found intragenic and intergenic haplotypes between *SFTPA1* and/or *SFTPA2* (42) and *SFTPB* and/or *SFTPD* haplotypes associated with risk or protective effect in RDS (43).

However, the impact of SNP-SNP interactions on RDS susceptibility has not been addressed before. The synergistic (epistatic) interactions among genetic variants of the surfactant proteins may alter disease susceptibility (44, 45), but this was not possible to study earlier due to the limitation of statistical approaches at the time. However, current more advanced statistical models may help identify the intricate epistatic interaction among multiple gene variants that play a significant role in multifactorial and complex diseases, such as RDS. Such analysis is likely to be beneficial to understand the impact of genetics on complex diseases, especially as we move toward personalized medicine.

In the present study, we studied intergenic and intragenic SNP-SNP interactions of the *SFTP* genes. We hypothesized that epistatic interactions among *SFTP* gene variants are associated with RDS susceptibility in preterm infants.

## Materials and Methods

### Study Samples

This study used available genotype data and clinical information in the Floros biobank at Penn State University, College of Medicine. These were collected and processed under an approved protocol by the institutional review board from the human subject protection office of the Pennsylvania State University (PSU) College of Medicine as well as the institutional review board of the respective centers where samples were collected in other Institutions other than PSU, as described previously (12, 29, 31, 32, 46, 47). The clinical and demographic data of the study samples are given in Table 1. The patients consisted of 848 preterm infants born <36 weeks of gestation, stratified by RDS, where 458 infants were diagnosed with RDS, and 477 infants did not develop RDS. RDS was diagnosed by clinical features of respiratory distress such as retractions, grunting, and flaring after birth. Chronic lung disease was diagnosed as needing supplemental oxygen at 28 days of life or 36 weeks postmenstrual age (50). Chorioamnionitis was diagnosed by clinical features such as maternal fever. The use of antenatal steroids was variable with betamethasone or dexamethasone.

**Table 1:** Clinical Characteristics of the cohort with and without RDS.

**Variables**	**No RDS (*n* = 458)**	**RDS (*n* = 477)**	***P*-value**
Gestational age (weeks): median (IQR)	33 (31, 35)	30 (26, 34)	<0.001^*^
Sex: *n* (%)			
Female	236 (51)	198 (41)	0.02^*^
Male	220 (48)	277 (58)	
Race: *n* (%)			
Non-Hispanic white	328 (71)	343 (72)	
Non-Hispanic black	64 (14)	82 (17)	0.09
Hispanic	20 (4)	25 (5)	
Asian-pacific islander	23 (5)	13 (2)	
Other/mixed parents	22 (4)	13 (2)	
Infant birth weight (g) ± SD	1,818 ± 515	1,474 ± 606	<0.001^*^
Preterm labor: *n* (%)			
Absent	64 (14)	74 (15)	0.36
Present	203 (44)	196 (41)	
Maternal diabetes mellitus: *n* (%)			
No	419 (92)	412 (94)	0.27
Yes	33 (7)	21 (5)	
Chorioamnionitis: *n* (%)			
No	161 (35)	204 (43)	0.26
Yes	35 (8)	33 (7)	
Antenatal steroid: *n* (%)			
No	1 (0.6%)	16 (3%)	0.0003^*^
Yes	280 (61%)	273 (57%)	
Surfactant use: *n* (%)			
No	448 (97)	167 (35)	<0.001^*^
Yes	8 (2)	305 (64)	
Chronic lung disease: *n* (%)			
No	297 (65)	238 (50)	<0.001^*^
Yes	16 (4)	92 (20)	

* The infants with RDS had younger gestational age at birth, lower birth weight, predominantly male, and had increased use of surfactant and higher incidence of chronic lung disease^**^.

The two groups (RDS, no RDS) did not differ in race, incidence of preterm labor, maternal diabetes mellitus, chorioamnionitis^***^.

^**^Chronic lung disease included infants treated with oxygen at 28 days of life or at 36 weeks postmenstrual age (48).

^***^Chorioamnionitis is diagnosed based on clinical features such as maternal fever (49).

A total of 17 SNPs of the SP genes *SFTPA1, SFTPA2, SFTPB, SFTPC*, and *SFTPD* were studied. These included five SNPs from *SFTPA1*: rs1059047, rs1136450, rs1136451, rs1059057, and rs4253527; four SNPs from *SFTPA2*: rs1059046, rs17886395, rs1965707, and 1965708; four SNPs from *SFTPB*: rs1130866, rs7316, rs2077079, and rs3024798; two SNPs from *SFTPC*: rs4715 and rs1124; and two SNPs from *SFTPD*: rs721917 and rs2243639. Polymerase chain reaction-restriction fragment length polymorphism (PCR-RFLP) was used to analyze the *SFTP* gene polymorphisms as described (49, 51, 52).

### Statistical Analysis

Wang et al. (53) developed a general multi-locus model for analyzing genetic associations in a case-control study. This model has three characteristics. First, it integrates classic quantitative genetic principles into a categorical data analysis framework, allowing epistatic interactions to be interpreted on a solid genetic basis. Second, this model can not only detect the genetic effects of single SNPs and pairwise genetic interactions, but also characterize high-order genetic interactions. That is, the model dissects genotypic differences into additive (a) and dominant (d) genetic effects at individual SNPs: additive × additive (aa), additive × dominant (ad), dominant × additive (da), and dominant × dominant (dd) epistatic effects at a pair of SNPs, and additive × additive × additive (aaa), additive × additive × dominant (aad), additive × dominant × additive (ada), additive × dominant × dominant (add), dominant × additive × additive (daa), dominant × additive × dominant (dad), dominant × dominant × additive (dda), dominant × dominant × dominant (ddd) epistatic effects at a triad of SNPs. Mounting evidence shows that high-order interactions play an important role in mediating complex traits and complex human diseases (54). Third, while the precise detection of a pairwise genetic interaction requires a huge number of samples, such as 5,000 (55), which may be hardly met in general studies, Wang et al.'s model is less sample size-reliant by coalescing case and control samples into a 2 × 2 contingency table for the detection of epistasis at any order. The statistical properties of Wang et al.'s model have been extensively studied through computer simulation, with results, presented in the original article, demonstrating its usefulness and robustness in a small-sample case-control study. Also, a detailed computational procedure of this model was given in the original article, allowing the readers to understand and repeat the model.

For each type of data analysis, case-control genotype observations were sorted into a 2 × 2 contingency table to test each of the genetic effects described above. For example, consider a SNP with three genotypes AA, Aa, and aa. To estimate its dominant effect, the effect size was compared to that of the heterozygote Aa against the average size of each of the two homozygotes AA and aa in cases and controls, respectively. Based on the resulting 2 × 2 contingency table, the logistic regression model was implemented to estimate the dominant effect of this SNP, and the effects were adjusted for age and sex. The odds ratio (OR) was estimated to assess the magnitude of the dominant/additive effect.

To estimate the additive effect, the size was compared as below,

Odds of genotype for cases = number of cases with AA/number of cases with aa

Odds of genotype for controls = number of controls with AA/number of controls with aa

OR = odds for cases/odds for controls

= $\frac{\left(\text{number of cases with AA}\times \text{number of controls with aa}\right)}{\left(\text{number of controls with AA}\times \text{number of cases with aa}\right)}$

For example-

OR = 1: Genotype difference is not associated with the disease;

OR > 1.0: Genotype AA is “more risky” (i.e., associated with higher risk for the disease than genotype aa)

OR < 1.0: Genotype aa is “more risky” for the disease than genotype AA

A similar procedure was applied to analyze all other genetic effects.

The significance of each effect was adjusted for multiple comparisons using the false discovery rate (FDR) controlled at 1%. Wang et al.'s simulation data indicate that a 100 × 100 sample size combination in an epistatic case-control model has a power of > 0.80 to detect significant associations in a 2 × 2 contingency table analysis (53). Thus, our current sample size provides adequate power to detect all the significant epistatic interactions.

## Results

### Clinical Characteristics of Infants With and Without RDS

Table 1 shows the demographic and clinical characteristics of infants with and without RDS. There were 458 infants without RDS and 477 infants who developed RDS. Infants with RDS were younger as assessed by gestational age at birth (30 vs. 33 weeks) and had lower birth weight (1,474 ± 606 gram vs. 1,818 ± 515 gram) compared to infants without RDS. Infants with RDS were predominantly male (58 vs. 48%, *p*-value 0.02). The two risk factors for RDS (gestational age and sex) were corrected in the analysis. Gestational age and birth weight are co-linear variables, and only one (gestational age) was chosen to be corrected in the analysis. As expected, infants who developed RDS had increased use of surfactant and a higher incidence of chronic lung disease than infants who did not have RDS. These outcomes are related to RDS rather than predictors (surfactant use and chronic lung disease); therefore, we did not correct them in the SNP-SNP interaction model. The use of antenatal steroids was significantly different between the two groups. However, ~40% of the antenatal steroid data were missing and may have caused bias in estimating this parameter.

### Association of *SFTP* SNP-SNP Interaction With RDS

#### Description

The associations of single SNP and intergenic/intragenic two and three SNP interactions with RDS are shown in Tables 2–4, respectively. The tables show the specific SNPs of the *SFTP* genes and their effect, either additive (a) or dominant (d). The additive effect of the SNP indicates that one of the homozygous alleles (one or two copies) is associated with the disease compared to the other homozygous allele. The dominant effect of the SNP indicates that the heterozygous genotype is associated with the disease compared to the mean of either homozygous genotype. The numbers 1, 2, or 3 are for SNP1, SNP2, or SNP3, respectively. For example, (a) a1d2 (Table 3) interaction means that the presence of any minor allele genotype of SNP1 and the heterozygous genotype of SNP2 is significant. (b) d1d2d3 (Table 4) interaction indicates that the combination of the heterozygous genotype at the first, second, and third SNP is associated with the disease.

**Table 2:** Single SNP associated with RDS.

**Gene**	**SNP**	**Effect**	**Odd ratio**	**95% CI**	***P*-value**	*P*-value Adjusted^*^
*SFTPA2*	rs17886395	Additive	0.16	0.06–0.43	0.0006	0.03

* *P-value is adjusted for gestational age, sex, as well as for multiple comparisons by FDR, P < 0.05*.

**Table 3:** The two SNP interactions associated with RDS susceptibility.

**Gene1**	**SNP 1**	**Gene2**	**SNP2**	**Effect**	**Odds ratio**	**95% CI**	***P*-value**	***P*-value adjusted**
*SFTPA2*	rs17886395	*SFTPD*	rs721917	d1d2	0.56	0.45–0.69	9.33E-08	9.77E-05
		*SFTPA1*	rs4253527	d1d2	1.69	1.32–2.07	8.88E-06	0.003097
*SFTPA1	rs1136450	*SFTPA1*	rs4253527	d1d2	1.77	1.42–2.19	3.08E-07	0.000161
				d1a2	0.54	0.41–0.72	2.91E-05	0.004226
*SFTPA2*	rs1965708	*SFTPA1*	rs1059047	d2	0.43	0.29–0.62	1.61E-05	0.004507
				d1d2	1.69	1.32–2.17	2.85E-05	0.004507
*SFTPB*	rs2077079	*SFTPC*	rs4715	a1d2	0.19	0.09–0.38	3.04E-05	0.004507
*SFTPB*	rs3024798			a1d2	5.7	2.56–12.65	3.44E-05	0.004507

Interaction effect: a- additive, d-dominant, ad-additive × dominant, dd-dominant × dominant between the two SNPs. The intragenic interaction is marked with an asterisk (*).

*The interactions associated with risk are highlighted in yellow*.

*Numbers 1 and 2 in the effect column represent SNP1 and SNP2, respectively*.

*The a1d2 stands for additive effect for SNP1 and dominant effect for SNP2*.

*The d1d2 stands for dominant effect for SNP1 and dominant effect for SNP2*.

*P-value is adjusted for gestational age, sex, and corrected for multiple comparisons by FDR, P-value adjusted <0.01*.

**Table 4:** Three SNP-SNP-SNP interactions of surfactant protein genes associated with RDS.

**Gene1**	**SNP1**	**Gene2**	**SNP2**	**Gene3**	**SNP3**	**Effect**	**Odd's ratio**	**95% CI**	***P*-value adjusted**
*SFTPA2	rs1059046	*SFTPA2*	rs1965707	*SFTPA2*	rs1965708	d1d2d3	0.55	0.46–0.65	7.74E-08
*SFTPA2*	rs1965707	*SFTPA2*	rs1965708	*SFTPA1*	rs1136450	d1d2d3	0.55	0.46–0.65	1.30E-07
						d1d3	1.92	1.47–2.51	0.001018
*SFTPA2*	rs1059046	*SFTPA2*	rs17886395	*SFTPA1*	rs1059047	d1d2d3	0.57	0.47–0.69	3.54E-05
						d1d2d3	0.59	0.49–0.72	0.000159
*SFTPA2*	rs17886395	*SFTPA2*	rs1965707	*SFTPA1*	rs1136451	d1d2d3	1.57	1.3–1.89	0.001033
*SFTPA2*	rs1059046	*SFTPA1*	rs1136451	*SFTPA1*	rs1059057	d1d2d3	0.54	0.44–0.65	8.12E-07
*SFTPA2*	rs17886395	*SFTPA1*	rs1059047	*SFTPA1*	rs1059057	a1d2d3	4.76	2.67–8.47	0.001024
*SFTPA2*	rs17886395	*SFTPA1*	rs1136450	*SFTPA1*	rs1059057	d1d2d3	0.57	0.47–0.69	0.000401
*SFTPA2*	rs17886395	*SFTPA1*	rs1059047	*SFTPA1*	rs1136450	d1d2d3	0.53	0.44–0.65	8.12E-07
*SFTPA2*	rs1059046	*SFTPA1*	rs1136450	*SFTPA1*	rs4253527	d1d2d3	1.53	1.28–1.81	0.001018
*SFTPA2*	rs17886395	*SFTPA1*	rs1059047	*SFTPA1*	rs1136451	d1d2d3	0.62	0.51–0.75	0.001235
*SFTPA1	rs1059047	*SFTPA1*	rs1136450	*SFTPA1*	rs1136451	d1d2d3	0.53	0.43–0.64	2.82E-07
*SFTPA1	rs1136450	*SFTPA1*	rs1136451	*SFTPA1*	rs1059057	d1d2d3	0.57	0.47–0.69	1.77E-05
*SFTPA1	rs1059047	*SFTPA1*	rs1136451	*SFTPA1*	rs1059057	d1a2d3	4.09	2.39–7.00	0.0012
*SFTPA2*	rs1059046	*SFTPD*	rs721917	*SFTPB*	rs7316	d1d2	0.53	0.41–0.67	0.000197
						d1d2a3	0.51	0.40–0.64	6.71E-05
*SFTPA1*	rs1136450	*SFTPA1*	rs4253527	*SFTPB*	rs7316	d1d2	2.01	1.56–2.60	6.71E-05
						d1d2a3	1.96	1.52–2.52	0.000196
*SFTPA2*	rs1965708	*SFTPD*	rs721917	*SFTPB*	rs1130866	d2d3	0.52	0.40–0.67	0.000362
*SFTPA2*	rs1059046	*SFTPA1*	rs4253527	*SFTPD*	rs721917	d1d3	0.51	0.40–0.64	3.54E-05
						d1a2d3	0.49	0.39–0.62	1.40E-05
*SFTPA2*	rs1059046	*SFTPA1*	rs1136450	*SFTPD*	rs721917	d1d2d3	0.53	0.45–0.63	1.12E-08
*SFTPA2*	rs17886395	*SFTPA1*	rs1136451	*SFTPD*	rs721917	d1d2d3	0.61	0.49–0.73	0.000467
*SFTPA2*	rs1965708	*SFTPA1*	rs1136450	*SFTPD*	rs2243639	d1d2d3	1.62	1.34–1.95	0.000273
*SFTPA2*	rs1965708	*SFTPA1*	rs1059057	*SFTPB*	rs2077079	d1d2d3	1.64	1.33–2.01	0.001275
*SFTPA2*	rs1059046	*SFTPB*	rs2077079	*SFTPB*	rs1130866	d1d2d3	0.67	0.57–0.79	0.001295
*SFTPB	rs2077079	*SFTPB*	rs3024798	*SFTPB*	rs7316	d1d2d3	0.63	0.52–0.76	0.001029

Interaction effect: a- additive, d-dominant, for example, dda-dominant × dominant × additive among the three SNPs. The intragenic interactions are marked with asterisks (*).

*The interactions associated with risk are highlighted in yellow*.

*Numbers 1, 2 and 3 in the effect column represent SNP1, SNP2, and SNP3, respectively*.

*The d1 stands for dominant effect for SNP1, d2 stands for dominant effect for SNP2, and a3 stands for additive effect for SNP3*.

*P-value adjusted is for gestational age, sex, and corrected for multiple comparisons by FDR, P < 0.01*.

#### Association of Single *SFTP* SNPs With RDS

Out of the 17 SNPs of the five *SFTP* genes, only the rs17886395 of the *SFTPA2* was associated by itself with RDS (Table 2). This SNP exhibited an additive effect on RDS susceptibility (OR 0.16, 95% CI 0.06–0.43, adjusted *p* = 0.03). This particular SNP is also noted to interact with other SNPs in the two and three SNP interactions models, as shown in Tables 3, 4. No other *SFTP* SNP by itself was associated with RDS at the adjusted value *p* < 0.01.

#### Association of Intragenic SNP-SNP Interactions With RDS in Two- and Three-SNP Interaction Model

##### Two SNP Model Intragenic Interactions

**Figure 1:**
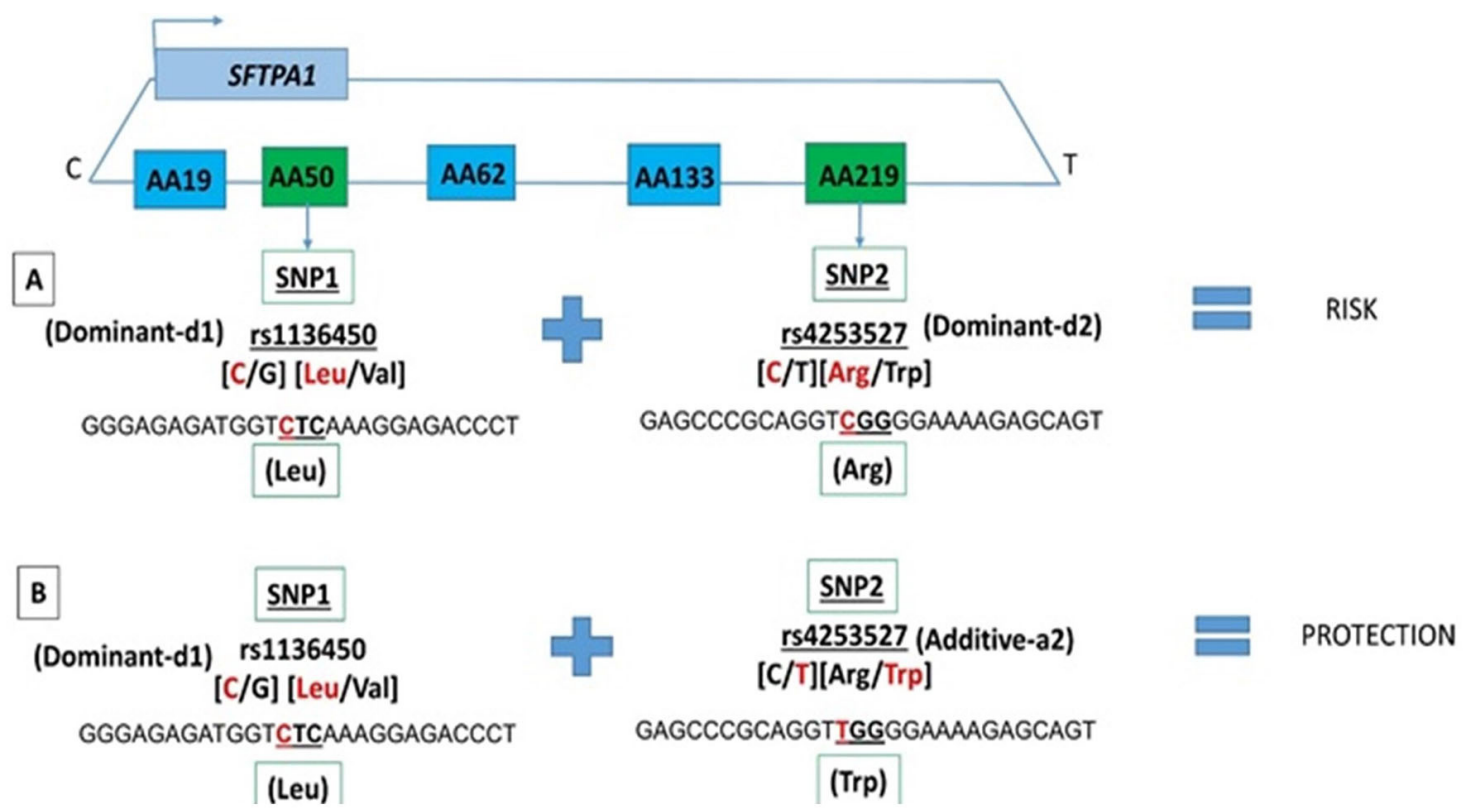
*SFTPA1* intragenic two SNP interactions and RDS susceptibility. It shows the schematic presentation of *SFTPA1* on the top and the arrow depicts the transcriptional orientation. The relative location of SNPs are shown from centromere (C) to telomere (T) and each box represents the amino acid number that includes the particular SNP. For example, AA50 denotes the rs1136450 SNP and AA219 denotes the rs4253527 SNP. The amino acid numbering is based on the precursor molecule and thus includes the signal peptide (56). In this two SNP intragenic interaction, underneath the green boxes are the SNP ID and the actual SNPs involved. **(A)** The dominant effect, dl of SNP1, is highlighted by red, as noted for the rs1134650 C variant that codes for leucine. This interacts with the dominant effect of SNP2, rs4253527 C variant that codes for arginine (in red) and increases risk of RDS. **(B)** The dominant effect, dl of SNP1, is highlighted by red and this SNP interacts with the additive effect of SNP2 rs4253527 T variant that codes for tryptophan and this interaction is protective for RDS.

Among the two SNP interactions, the only intragenic interaction included *SFTPA1* SNPs; rs1136450 and rs4253527 (Table 3), and this combination exhibited two effects, where the d1d2 interaction was associated with increased risk for RDS (OR 1.77, 96% CI 1.42–2.19, adjusted *P* = 0.0001), and the d1a2 was associated with protection for RDS (OR 0.54, 95% CI 0.41–0.72, adjusted *P* = 0.004) (Figure 1).

##### Three SNP Model Intragenic Interactions

There were five intragenic interactions associated with RDS. Three interactions were among SNPs of the *SFTPA1* and two involved the *SFTPA2* and *SFTPB* genes. The *SFTPA2* SNPs: rs1059046, rs1965707, and rs1965708 exhibited an effect, d1d2d3, that was protective for RDS (OR = 0.55, 95% CI 0.46–0.55, adjusted *p* < 0.01). The *SFTPA1* gene variants: rs1059047 (SNP1), rs1136451 (SNP2), rs1059057 (SNP3) in a three-SNP interaction (d1a2d3) increased the risk for RDS (OR 4.09, 95% CI 2.39–7.00, adjusted *p* = 0.0012) (Table 3). The other intragenic interaction, d1d2d3, was found among *SFTPB* SNPs: rs2077079 (SNP1), rs3024798 (SNP2), and rs7316 (SNP3), as d1d2d3, and this was protective for RDS (OR = 0.63, 95% CI 0.52–0.76, adjusted P 0.001).

#### Association of Intergenic Interactions Among the Surfactant Protein Genes SNPs With RDS in a Two- and Three-SNP Model

##### Two SNP Model Intergenic Interactions

The two SNP interactions are shown in Table 3. The combination of *SFTPA2* rs17886395 (SNP1) with (i) *SFTPA1* rs4253527 (SNP2) as d1d2, increased risk of RDS (OR 1.69, 95% CI 1.32–2.17, adjusted *p* = 0.004), and (ii) *SFTPA1* rs1059047 (SNP2) as d2 without any epistatic effect from SNP1 was protective (OR 0.43, 95% CI 0.29–0.62, adjusted *p* = 0.004). The *SFTPA2* SNP rs17886395 interaction with the *SFTPD* SNP rs721917 was protective when both had a dominant effect (OR 0.56, 95% CI 0.45–0.69 adjusted *p* < 0.01). Intergenic SNP-SNP interactions were also noted between each of the two of the *SFTPB* SNPs (rs2077079 or rs3024798) and one *SFTPC* SNP rs4715 associated with protection or risk against RDS, as shown in Table 3.

##### Three SNP Model Intergenic Interactions

Table 4 shows the intergenic three SNP interactions of the *SFTP* genes associated with RDS. There were a total of 28 intergenic interactions. There were four *SFTPA2* SNPs studied. Among them, the rs17886395 SNP, found to have an additive effect and be protective for RDS by itself in the single SNP model, was present in 7 out of the 28 intergenic interactions and in 5 out of the 7 interactions were noted to be protective.

**Figure 2:**
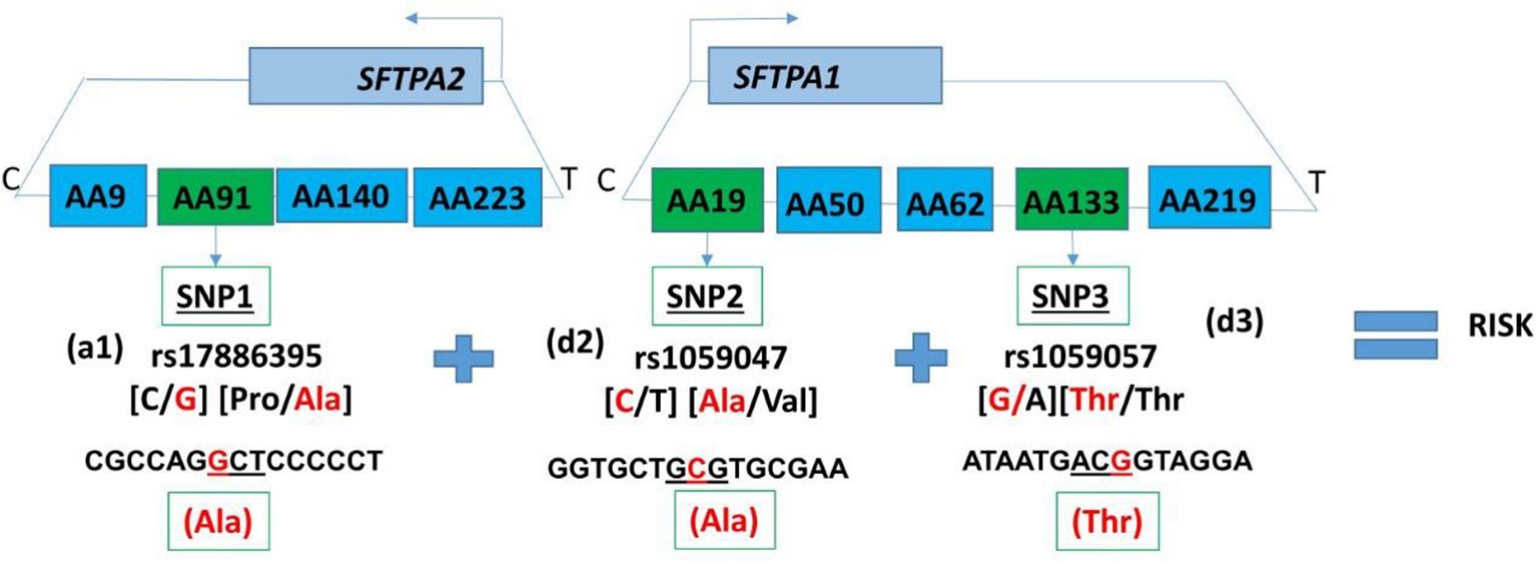
Intergenic three SNP interaction and RDS susceptibility. It shows the schematic presentation of *SFTPA2* and *SFTPA1* on the top and the arrows depict the opposite transcriptional orientation. The relative location of SNPs is shown from centromere (C) to telomere (T) and each box represents the amino acid number that includes the particular SNP. For example, AA91 denotes the rs17886395 SNP, AA19 denotes the rs1059047 SNP, and AA133 denotes the rs1059057 SNP. In this three SNP intergenic interaction, underneath the green boxes are the SNP ID and the SNPs involved. The additive effect of SNP1, rs17886395 G variant that codes for alanine (highlighted in red) interacts with SNP2 and SNP3 of *SFTPA1* in a dominant effect and increases risk of RDS.

The five *SFTPA1* gene SNPs exhibited mainly a dominant effect. The rs1136450 was involved in the highest number of interactions (10 intergenic interactions), and the other *SFTPA1* SNPs had fewer than 5 interactions showing either protective or risk effect. An example of a three intergenic SNP interaction is shown diagrammatically in Figure 2. This figure depicts an interaction among three SNPs of *SFTPA1* and *SFTPA2*. In this intergenic interaction, the additive effect of SNP1, rs17886395, G variant that codes for alanine interacts with SNP2 (rs1059047) and SNP3 (rs1059057) of *SFTPA1* in a dominant effect. This interaction, based on odd's ratios, is associated with increased disease susceptibility. It has the highest odd's ratio (OR 4.76, 95% CI 2.67–8.47) compared to the odd's ratios of the other three SNP interactions.

The *SFTPB* SNPs (rs7316, rs1130866, rs2077079) were involved in 5 intergenic interactions, and the *SFTPD* SNPs (rs721917, rs2243639) were involved in a total of 6 intergenic interactions, and they were mainly in a dominant effect.

### Hydrophobic vs. Hydrophilic Surfactant Protein Gene SNP Interactions

**Figure 3:**
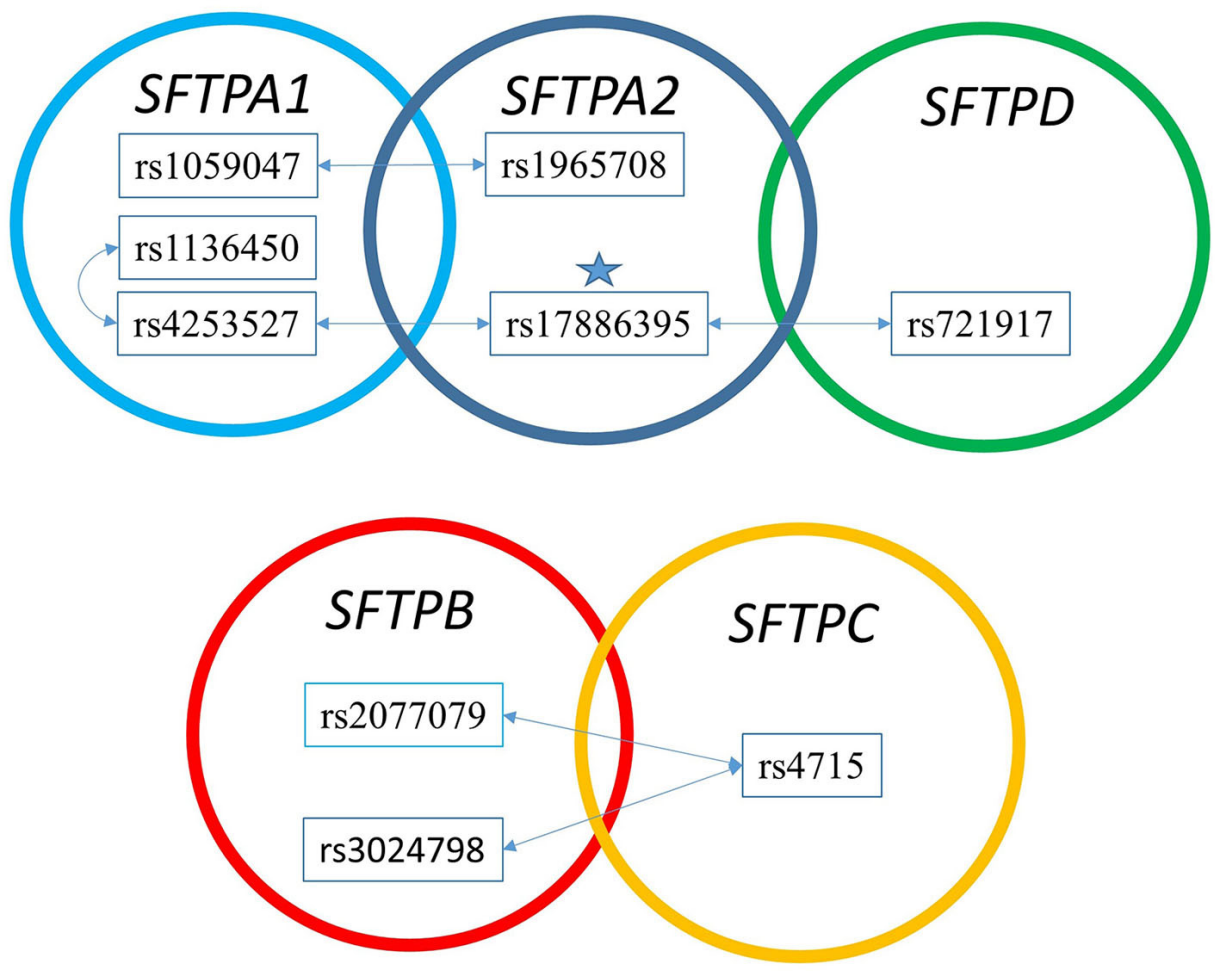
The two SNP interaction in RDS susceptibility. Associations between RDS and the two SNP-SNP interactions are shown. The star marks the *SFTPA2* SNP shown to associate with RDS by itself. **(A)** depicts the two SNP-SNP intergenic and intragenic interactions of the hydrophilic SP genes associated with RDS. **(B)** depicts the two SNP-SNP intergenic and intragenic interactions of the hydrophobic SP genes associated with RDS.

**Figure 4:**
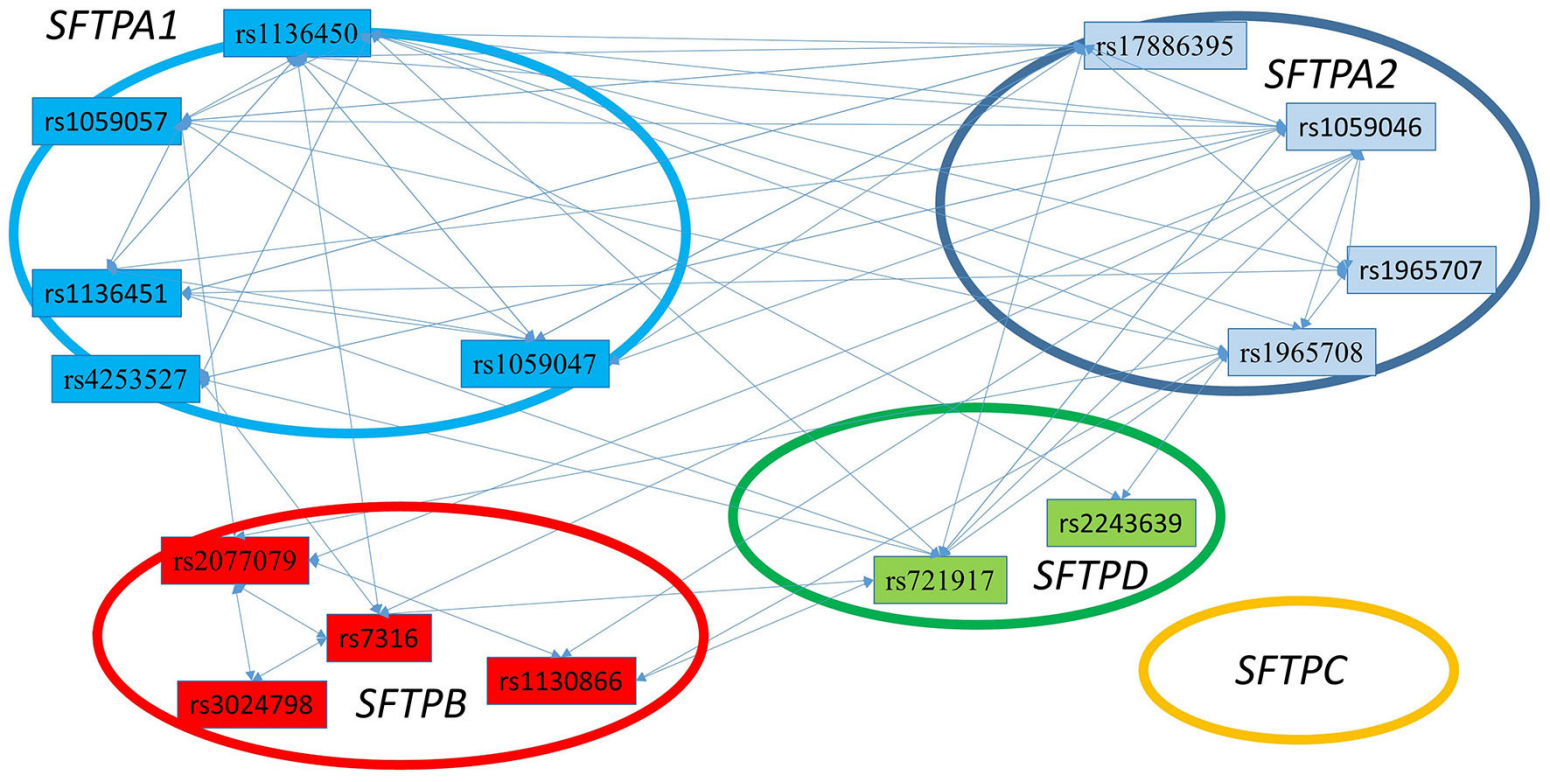
The three SNP interactions associated with RDS susceptibility. The figure depicts intergenic and intragenic interactions of *SFTPA1, SFTPA2, SFTPD*, and *SFTPB* genes. No three SNP interactions were observed that involved *SFTPC* SNPs. There are a total of 28 three SNP interactions. All interactions (but one) involved *SFTPA1* and/or *SFTPA2*.

Figure 3 shows that the SNPs of the hydrophobic *SFTPB* and *SFTPC* interacted with each other in the two-SNP model, and the SNPs of the hydrophilic *SFTPA1, SFTPA2*, and *SFTPD* SNPs also interacted with each other. There was no interaction between any of the hydrophobic and the hydrophilic SPs SNPs. The three-SNP model (Figure 4) depicted an intricate network of interactions among all the *SFTP* genes, except for *SFTPC*. A total of 28 three SNP interactions were identified. The *SFTPA1* and *SFTPA2* have the maximum number of interactions and, along with *SFTPD*, interacted with *SFTPB*. All three SNP interactions, except for one intragenic interaction of *SFTPB* (rs2077079-SNP1, rs3024798-SNP2, rs7316-SNP3 as d1d2d3), involved either *SFTPA1* or *SFTPA2*. This highlights the impact and importance of *SFTPA1* and *SFTPA2* in RDS.

## Discussion

Although *SFTP* variants have been implicated in RDS (10, 27, 39), the statistical method used at the time had a limited ability to detect complex epistatic interactions among multiple SNPs. However, a more recent methodology by Wang et al. (53) enables investigation of complex SNP-SNP interactions by employing SNP interaction models. As one of very few statistical models that can analyze high-order interactions, Wang et al.'s (53) model has been used in a variety of case-control studies, showing its elegance and robustness. For example, using this model, interactions among *SFTP* SNPs were detected to impact cystic fibrosis (57), pediatric acute respiratory failure (58), and hypersensitivity pneumonitis (59). In this study, we used 17 well-studied SNPs of *SFTPA1, SFTPA2, SFTPB, SFTPC*, and *SFTPD* to investigate the impact of individual SNPs and SNP interactions among SNPs in the same gene or between two or among three different genes. This approach revealed that (a) the highest number of the two and three SNP interactions were among SNPs of the *SFTPA1* and *SFTPA2*, and these were associated with risk or protection for RDS. (b) Only the rs17886395 (C allele) of the *SFTPA2* was protective for RDS in a single-SNP model. (c) In the two SNP models, there was no interaction between the hydrophilic SFTPA1, SFTPA2, *SFTPD* SNPs, and the hydrophobic *SFTPB* or *SFTPC* SNPs. (d) the three SNP models showed intricate intergenic and intragenic interactions among SNPs of the *SFTPA1, SFTPA2, SFTPB*, and *SFTPD;* however, *SFTPC* did not interact with any of the other *SFTP* SNPs. Thus, in the present study we show not only association of a single SNP but also of two and three SNP interactions to associate with RDS susceptibility.

### Association of an *SFTPA2* SNP With RDS in a Single-SNP Model

Using the stringent criteria of FDR correction with 1% (*p* < 0.01), none of the single *SFTP* SNPs was associated with RDS. When the FDR correction was set at 5% (*p* < 0.05), the rs17886395 G allele of the *SFTPA2* gene exhibited an additive effect and increased risk for neonatal RDS compared to the C allele. The 1A^3^ haplotype that includes the G allele increased the risk of TB in Mexicans (60). However, the C allele of the same SNP, found to be protective of RDS (present study), has also been protective against infection, such as RSV in Finnish infants (61). In contrast, in an Ethiopian study group, the C allele was associated with increased risk of TB (62), and this allele as part of 6A/1A genotype was associated with risk in community-acquired pneumonia in a Spanish study group (63). Several haplotypes of *SFTPA1* and *SFTPA2* have been well-characterized (39, 64) and the most common haplotype, 6A^2/^1A^0^, has been associated with low SP-A protein expression in a study of patients with sudden infant death syndrome (65). It is of interest that the C allele of the rs17886395 SNP in pediatric diseases (i.e., RDS, RSV) is associated with protection, but in diseases likely to occur in adults (i.e., TB, community-acquired pneumonia) is associated with risk. Whether disease susceptibility by the C allele of the rs17886395 SNP is influenced by the lung environment in an age-dependent manner remains to be determined. The association of this particular SNP (rs17886395) in RDS susceptibility in the current study may not be surprising. Infection is a common complication of RDS and prematurity, and therefore the alleles of this SNP may differentially affect disease susceptibility.

The rs17886395 (C/G) is located in the collagen-like domain of *SFTPA2* and changes the encoded amino acid Pro/Ala at codon 91 (41). It has been shown that proline normally stabilizes collagen triple helices due to conformational restrictions of the pyrrolidine ring and the presence of tertiary amides, while alanine substitutions tend to destabilize the triple helix (66). Thus, the G allele/*G*CT encoding alanine may destabilize the structure and explain the risk susceptibility.

### Association of *SFTP* SNPs With RDS in a Two-SNP Model

We observed an association of the intragenic interaction between two SNPs (rs1136450 and rs4253527) of the *SFTPA1* with RDS susceptibility in the two-SNP model. The susceptibility of RDS changes based on the effect of rs4253527 in that interaction, i.e., dominant and additive effect of rs4253527 is associated with increased and decreased risk of RDS, respectively (Figure 1). This indicates that an additive or a dominant effect of the same SNP may change the susceptibility of an individual to a particular disease based on interactions with other SNPs. The rs4253527 (C/T) is located within the carbohydrate recognition domain (CRD) of the *SFTPA1* and changes the amino acid arginine (CGG) to tryptophan (TGG) at amino acid 219. This change may differentially affect innate immune processes under various conditions, including oxidative stress, because tryptophan is more sensitive to oxidation than arginine (56, 67). *SFTPA1* variants that differ in CRD at rs4253527 have been shown to differ in their ability to enhance phagocytosis (68) and cytokine production (69). Moreover, the CRD of surfactant proteins A and D are known to mediate binding to infectious agents such as *Pneumocystis carinii* (70, 71) and therefore the susceptibility to RDS may be interconnected with response to infection. The rs1136450 (C/G) SNP has a leucine (CTC) to valine (CTC) substitution at amino acid 50 and together with rs4253527, may impact protein function, but direct experimental evidence is lacking. Moreover, *SFTPA1* has been shown to more efficiently affect surfactant reorganization (than *SFTPA2*) in the alveolar space and inhibit surfactant inactivation by serum proteins (25). However, considering the complexity of *SFTPA* variants and their potential contribution to health and disease status, it is conceivable that the activity of a gene product in a given microenvironment, such as that in prematurity, is altered, and this may variably affect the health of the individual.

There were no significant interactions observed between SNPs of the hydrophilic and hydrophobic SPs. In contrast, previous observations have shown an association of *SFTPB* and *SFTPA1* and/or *SFTPA2* with increased risk of neonatal RDS in case-control studies (32, 34, 36). These apparent contrasting findings could be due to differences in the patient population, sample size, and/or statistical approaches used in previously reported studies and the present study.

### Association of *SFTP* SNPs With RDS in a Three-SNP Model

This study, to our knowledge, is the first to show that interactions among three SNPs of the SP genes and their epistatic effect associate with RDS susceptibility. The majority of prior studies have at most reported interactions between two SNPs of the SP genes. The three SNP models in the present study showed that the highest number of intergenic and intragenic interactions involved *SFTPA1* and *SFTPA2*, indicating perhaps the importance of these genes in RDS.

#### An SFTPA1 SNP Is Involved in the Highest Number of the Three-SNP Interactions

The SNP rs1136450 with a dominant effect had the highest number of interactions (*n* = 9), and these were associated with either risk or protection for RDS. The rs1136450 (C/G) results in an amino acid change, Leu/Val (CTC/GTC) at codon 50 (39, 41). This SNP is located in the N-terminal collagen region and the change in amino acid may affect the binding to receptors such as calreticulin/CD91 on phagocytes (72–74). The G allele (valine) of this SNP is associated with risk of interstitial pulmonary fibrosis (IPF) in a Mexican study group (49). On the other hand, the same allele was protective in community-acquired pneumonia (63). In prior studies, this allele has been associated with risk for RDS in Finnish, whites, and blacks (29, 30); however, this was not seen in a Korean study group (75). The current study showed that this SNP had a risk or protective effect based on interactions with SNPs of other *SFTP* genes. The various interactions may change the qualitative and/or quantitative function of *SFTPA*, and this could explain the variable outcome.

#### SFTPA2 SNPs Are Involved in the Three-SNP Interactions

The rs1059046 SNP of *SFTPA2* was also found to have a high number of interactions (*n* = 8), and all of the interactions with a dominant effect were shown to be protective for RDS. This SNP changes the amino acid Asn/Thr at codon 9 (AAC/ACC). This amino acid is part of the signal peptide and may affect the processing of SP-A2. The A allele of this SNP of *SFTPA2* was also noted to have a protective role in community-acquired pneumonia (63). Of note, prior studies have shown the A allele, either in its homozygous or heterozygous form to be associated with risk for the respiratory syncytial virus (RSV) (61, 76) as well as influenza (77). The rs17886395 of *SFTPA2*, which was described in detail above, was also noted to have a high number of three SNP interactions (*n* = 7), five of them had a dominant effect with a protective role and the remaining two (dominant or additive) were associated with risk in RDS. These together highlight the complexity of SNP interactions and their important effect on disease susceptibility.

#### SFTPB SNPs Are Involved in the Three-SNP Interactions

There was one significant intragenic interaction (rs2077079, rs3024798, and rs7316). Each SNP exhibited a dominant effect and this interaction was associated with decreased risk of RDS. The rs2077079 (C/A) is located 10 nt downstream of the TATAA box, 5′ regulatory region and may affect gene transcription. The rs3024798 (A/C) is located at the splice sequence of intron 2-exon 3 and may affect splicing. The rs7316 (A/G) is located in the 3′UTR, at 4 nt upstream of the TAATAAA polyadenylation signal and may affect polyadenylation (78). The location of these SNPs indicates that these may affect the processing and/or regulation of SP-B. Whether any of these mechanisms are negatively affected in RDS remains to be determined. However, each of these three SNPs has been previously shown to associate with various lung diseases (29, 57, 79, 80). The A allele of rs2077079 is associated with risk of RDS in blacks, whereas the A allele of rs3024798 is associated with protection of RDS (29). The A allele of rs7316 is associated with risk of RDS (79) and acute lung injury in African-American children (80). However, the dominant effect of rs7316 is associated with mild CF (57). It is interesting that these SNPs by themselves have been associated with risk or protection of RDS; however, the present study highlights the importance of SNP interactions, as these could mediate a differential epistatic effect compared to individual SNPs and that this may have a significant effect on the actual health/disease outcome of an individual under certain conditions.

#### SFTPC SNPs Were Not Involved in the Three-SNP Interactions

None of the SNPs were identified in the three SNP model, even though single *SFTPC* SNPs have been associated with RDS (38, 81) and other pulmonary diseases such as interstitial lung disease (82). The hydrophobic *SFTPB* and *SFTPC* SNPs showed significant interactions in the two SNP model but not in the three SNP model. Furthermore, the two *SFTPC* SNPs rs1124 and rs4715 change amino acids 186 and 138, respectively. Although their effect on the functional or structural integrity of SP-C is not known, these likely affect processing of the precursor SP-C molecule rather than the mature SP-C, because these amino acids are part of the SP-C precursor and not of the mature SP-C.

#### SFTPD SNPs Are Involved in the Three-SNP Interactions

The *SFTPD* SNPs were involved in intergenic interactions associated with RDS susceptibility. The *SFTPD* rs721917 (C/T) SNP changes Threonine (C) to Methionine (T) at position 11 in the mature protein. The C allele of the rs721917 SNP, is associated with O- linked glycosylation of threonine leading to a partial posttranslational modification and this may alter the tendency to form multimers (83, 84). Moreover, this SNP is associated with SP-D levels, with the T allele (methionine) being correlated with increased levels (83–85). The T allele of this SNP was protective for RDS (86, 87), whereas some studies reported no association with RDS (88). The current study also supports previous observations where *SFTPA2* and *SFTPD* haplotypes were shown to be protective against RDS (42).

Although the present study has a relatively large sample size, one limitation is that the patient population differs from that of the controls in terms of age, birth weight, and sex. However, the analyses were adjusted for age and sex (birth weight was not corrected due to collinearity with gestational age). Another study limitation may be reduced generalizability as both study groups were predominantly whites. It is also possible that we have missed some significant interactions due to the use of stringent criteria such as those imposed by the FDR correction, set at 1% to avoid spurious associations. Nonetheless, the present findings need to be replicated. The SNP interactions and their association with the disease phenotype may be affected by the severity of RDS, which was not captured in this study. Around 40% of the data on important parameters such as antenatal use of steroids were missing and that may have introduced bias in the estimation of the difference between groups. The diagnosis of chronic lung disease included oxygen use at 28 days or oxygen at 36 weeks postmenstrual age. The definition for BPD has evolved over time and hence the study characteristic does not capture the current definition of BPD, consistently, as per NICHD 2019 (89).

Despite the above limitations, this study indicates a greater role of *SFTPA1* and *SFTPA2* in RDS susceptibility as they had the most interactions with SNPs of other SFTPs in the two and three-SNP models. Furthermore, the concern for infection in the setting of prematurity and chorioamnionitis sets up the *SFTPA1* and *SFTPA2* gene products, SP-A1 and SP-A2, as very important molecules for the first line of defense and regulation of various processes of the alveolar macrophage (17). Our animal studies, among others, have shown that SP-A1 and SP-A2 regulate the miRNome of the alveolar macrophage (90) and the alveolar epithelial type II cells in response to ozone exposure (91). Most importantly, these differentially affect survival in response to infection in young and old mice (92, 93) and lung function (26). Of interest, the commercially available exogenous surfactant preparations used to treat RDS, lack SP-A (94) (they only have SP-B and S-PC), but yet infection is a major comorbidity with RDS.

Furthermore, surfactant lipids and SP-A exhibit anti- and pro-inflammatory effects, respectively, on immune cells under baseline conditions, and surfactant lipids have been shown to attenuate the SP-A effect (13, 95, 96). Thus, the absence of SP-A in the exogenous surfactant preparations and the additional surfactant lipids provided by the exogenous preparation may negatively contribute to a further imbalance of pro and anti-inflammatory processes (95) in the premature lungs. With ongoing trials of SP-A peptides to treat asthma and the use of SP-A peptides to treat RSV (97–99) the present findings point to a future need to investigate SP-A as adjunct therapeutic modality for RDS as well.

## Data Availability Statement

The data analyzed in this study are subject to the following licenses/restrictions: the de-identified dataset is part of the FLOROS biobank at the Penn State University, College of Medicine. Requests to access these datasets should be directed to Joanna FLoros, Jfloros@psu.edu.

## Ethics Statement

The studies involving human participants were reviewed and approved by Institutional Research Board (IRB) at Penn State University, College of Medicine. Written informed consent to participate in this study was provided by the participants' legal guardian/next of kin.

## Author Contributions

SA: data curation. MY, LY, and RW: formal analysis. JF: funding acquisition. BN and JF: resources. RW and JF: supervision and writing—review and editing. SA, CG, and JF: writing—original draft. All authors read and approved the final manuscript.

## Funding

This study was supported by NIH grant R37 HL34788 to JF.

## Conflict of Interest

The authors declare that the research was conducted in the absence of any commercial or financial relationships that could be construed as a potential conflict of interest.

## Publisher's Note

All claims expressed in this article are solely those of the authors and do not necessarily represent those of their affiliated organizations, or those of the publisher, the editors and the reviewers. Any product that may be evaluated in this article, or claim that may be made by its manufacturer, is not guaranteed or endorsed by the publisher.

## Abbreviations

SNP: Single nucleotide polymorphism
RDS: respiratory distress syndrome
*SFTP*: surfactant protein gene
BW: birth weight
SP: surfactant protein.
