# Corrigendum: Single nucleotide polymorphisms interactions of the surfactant protein genes associated with respiratory distress syndrome susceptibility in preterm infants

**DOI:** 10.3389/fped.2022.1069526

**Published:** 2022-11-30

**Authors:** Shaili Amatya, Meixia Ye, Lili Yang, Chintan K. Gandhi, Rongling Wu, Beth Nagourney, Joanna Floros

**Affiliations:** ^1^ Department of Pediatrics, Center for Host Defense, Inflammation, and Lung Disease (CHILD) Research, Pennsylvania State University College of Medicine, Hershey, PA, United States; ^2^ Center for Computational Biology, College of Biological Sciences and Technology, Beijing Forestry University, Beijing, China; ^3^ School of First Clinical Medicine, Nanjing University of Chinese Medicine, Nanjing, China; ^4^ Public Health Science, Pennsylvania State University College of Medicine, Hershey, PA, United States; ^5^ Albert Einstein College of Medicine, New York, NY, United States; ^6^ Obstetrics and Gynecology, Pennsylvania State University College of Medicine, Hershey, PA, United States

**Keywords:** epistasis, neonatal, genetic variants, pulmonary, allele

A Corrigendum on Single nucleotide polymorphisms interactions of the surfactant protein genes associated with respiratory distress syndrome susceptibility in preterm infants By Amatya S, Ye M, Yang L, Gandhi CK, Wu R, Nagourney B and Floros J. (2021). Front. Pediatr. 9: 682160. doi: 10.3389/fped.2021.682160

In the published article, there was an error in Figure 2 pertaining to SNP2 and SNP3 and is limited to the last 3 lines shown under each of these SNPs. The corrected Figure 2 appears below.

In the published article, there was an error in the **Results** section, subsection “Association of SFTP SNP-SNP Interaction With RDS”, subsection “Three SNP model intergenic interactions”, where “SNP 3- 1059047” should be “SNP3- 1059057”.

This sentence previously stated:

“This figure depicts an interaction among three SNPs of *SFTPA1* and *SFTPA2*. In this intergenic interaction, the additive effect of SNP1, rs17886395, G variant that codes for alanine interacts with SNP2 (rs1059047) and SNP3 (rs1059047) of *SFTPA1* in a dominant effect.”

The corrected sentence appears below:

“This figure depicts an interaction among three SNPs of *SFTPA1* and *SFTPA2*. In this intergenic interaction, the additive effect of SNP1, rs17886395, G variant that codes for alanine interacts with SNP2 (rs1059047) and SNP3 (rs1059057) of *SFTPA1* in a dominant effect.”

The authors apologize for this error and state that this does not change the scientific conclusions of the article in any way. The original article has been updated.

**Figure 2:**
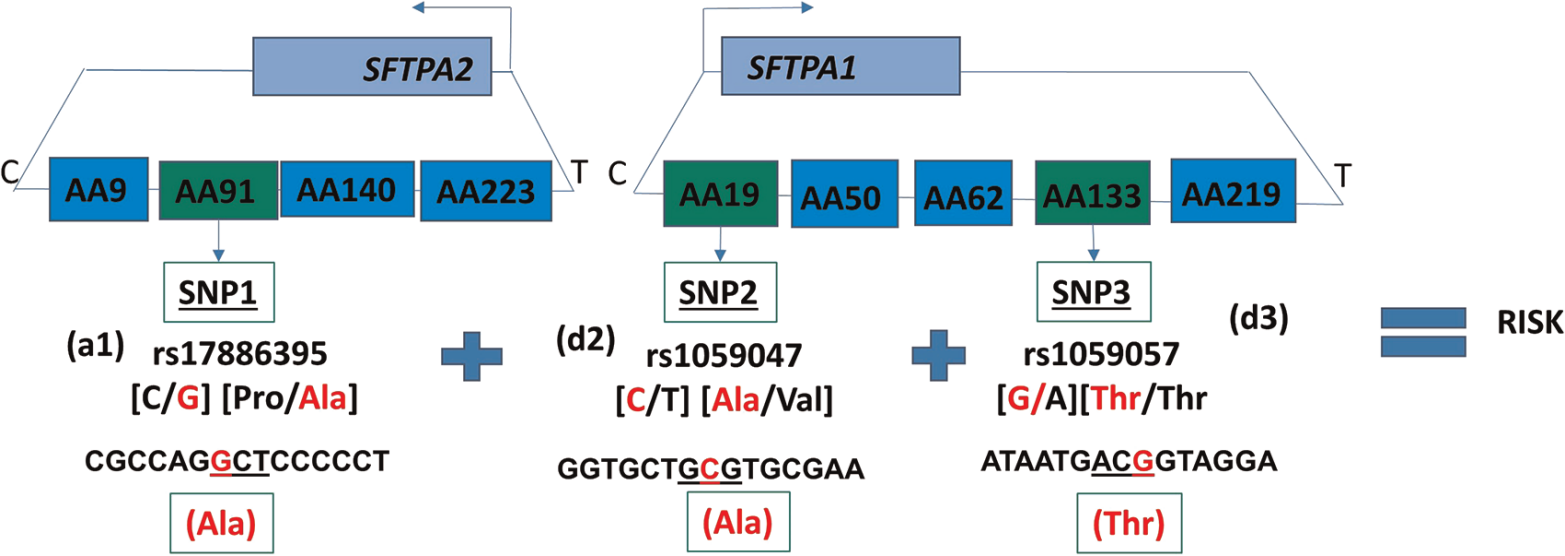
Intergenic three SNP interaction and RDS susceptibility. It shows the schematic presentation of *SFTPA2* and *SFTPA1* on the top and the arrows depict the opposite transcriptional orientation. The relative location of SNPs is shown from centromere (C) to telomere (T) and each box represents the amino acid number that includes the particular SNP. For example, AA91 denotes the rs17886395 SNP, AA19 denotes the rs1059047 SNP, and AA133 denotes the rs1059057 SNP. In this three SNP intergenic interaction, underneath the green boxes are the SNP ID and the SNPs involved. The additive effect of SNP1, rs17886395G variant that codes for alanine (highlighted in red) interacts with SNP2 and SNP3 of *SFTPA1* in a dominant effect and increases risk of RDS.

